# New adaptive laboratory evolution database highlights the need for consolidating directed evolution data

**DOI:** 10.1093/synbio/ysz009

**Published:** 2019-03-25

**Authors:** Bojar Daniel

**Affiliations:** 4058 Basel, Switzerland

*Synthetic Biology*, Volume 4, Issue 1, 1 January 2019, https://doi.org/10.1093/synbio/ysz004

Published: 04 February 2019

The affiliation was changed as there was a technical problem with the departmental affiliation of the main author.

